# A Narrative Review of the Therapeutic Effectiveness of Lenvatinib in Comparison With Immunotherapy for the Treatment of Non-Viral Advanced Hepatocellular Carcinoma

**DOI:** 10.7759/cureus.105157

**Published:** 2026-03-13

**Authors:** Pankaj Sonone, Sumit Goyal, Nilesh Lokeshwar, Shruti Dharmadhikari, Gaurav Puppalwar, Chintan Khandhedia, Amey Mane, Suyog Mehta

**Affiliations:** 1 Medical Affairs and Clinical Research, Sun Pharma Laboratories Limited, Mumbai, IND; 2 Medical Oncology, Rajiv Gandhi Cancer Institute and Research Centre, New Delhi, IND; 3 Medical Oncology, Lilavati Hospital and Research Centre, Mumbai, IND

**Keywords:** advanced hepatocellular carcinoma, clinical studies, immunotherapy, lenvatinib, non-viral etiology

## Abstract

The therapeutic landscape of advanced hepatocellular carcinoma (aHCC) has evolved with the advent of targeted therapies and immune checkpoint inhibitors (ICIs). While ICI-based regimens such as atezolizumab and bevacizumab are widely adopted as first-line therapy, emerging evidence indicates reduced effectiveness in patients with non-viral etiologies such as metabolic dysfunction-associated steatohepatitis (MASH) and steatotic liver disease (MASLD). With viral HCC declining and non-viral cases increasing, lenvatinib, a potent multi-kinase inhibitor, has gained attention for its favorable efficacy in this subgroup. A thorough literature search was conducted across PubMed, MEDLINE (Medical Literature Analysis and Retrieval System Online), Google Scholar, Web of Science, and Science Direct, for English-language studies published from 2014 to 2025. Relevant randomized controlled trials, observational studies, real-world evidence, and registered clinical trials were reviewed. Our review indicates that lenvatinib may outperform ICI-based regimens in overall survival (OS) and progression-free survival (PFS) among patients with non-viral aHCC, particularly those with MASLD/MASH; however, this observation is based predominantly on retrospective studies. Its mechanisms, including angiogenesis inhibition and immune modulation, offer advantages in the immunosuppressive tumor microenvironment of non-viral HCC. Safety data suggest a manageable profile, with adverse events comparable to those in viral HCC. Emerging data also support lenvatinib-based combination therapies to enhance efficacy. Moreover, we have also discussed the existing challenges in managing HCC in clinical practice. Lenvatinib is a promising first-line option in non-viral aHCC in patients with MASH or MASLD. Given the etiology-specific response to therapy, future research and clinical guidelines should consider stratified approaches. Evidence suggests superior survival outcomes compared with ICIs. Etiology-specific responses highlight the need for stratified therapeutic approaches and consideration of lenvatinib-based combination therapies.

## Introduction and background

Hepatocellular carcinoma (HCC) is one of the most lethal malignancies, accounting for 85-90% of primary liver cancers globally [1], and accounting for over 38,703 new cases and 36,953 deaths annually in India [2]. Although hepatitis B virus (HBV) and hepatitis C virus (HCV) have historically dominated HCC etiology, widespread HBV vaccination and effective antiviral therapies have substantially reduced virus-related disease [3]. HBV vaccination alone has lowered new HBV-related HCC cases by an estimated 83% [4], with hepatitis B surface antigen (HBsAg) prevalence in young children falling from 4.7% to 1.5% [5], and chronic HCV incidence is projected to continue declining from 227 to 198 cases per one million individuals [4].

In contrast, non-viral advanced HCC (aHCC) is rising globally. Between 1990 and 2019, HBV-related HCC decreased from 53% to 42%, while metabolic dysfunction-associated steatohepatitis (MASH)- and alcohol-related HCC increased from 5% to 6% and 13% to 18%, respectively [6]. Indian data similarly show that metabolic dysfunction-associated steatotic liver disease (MASLD) is the leading cause of HCC (35.5%), followed by HBV, HCV, and heavy alcohol use [7]. With obesity, MASLD, alcohol use, and diabetes on the rise, non-viral aHCC has become increasingly common [3,8].

Therapies such as tyrosine kinase inhibitors, immune checkpoint inhibitors, and agents targeting vascular endothelial growth factor/fibroblast growth factor signaling, estimated glomerular filtration rate (eGFR), mechanistic target of rapamycin (mTOR), and hepatocyte growth factor (HGF)/mesenchymal‐epithelial transition (c‐MET) aim to modulate the underlying chromosomal instability, immune imbalance, and epigenetic changes seen in non-viral aHCC [9-14].

Sorafenib was the first approved multi-kinase inhibitor (MKI) for non-viral aHCC [15], followed by lenvatinib after the REFLECT trial [16,17]. Although immune checkpoint inhibitor (ICI)-based therapies have been potential treatment options, including atezolizumab-bevacizumab [18], several studies reported reduced ICI-based regimen effectiveness in MASLD/MASH-associated HCC compared with viral etiologies [19-21]. Emerging evidence suggests lenvatinib may be particularly relevant in non-viral aHCC [14,22], yet its role, especially in MASLD/MASH, remains under-characterized.

This narrative review, therefore, contextualizes the epidemiological shift toward metabolic-related HCC, outlines its biological and clinical challenges, and evaluates the therapeutic potential of lenvatinib, both as monotherapy and in combination with other treatments, through reviewing multiple studies on non-viral aHCC, and also compares the efficacy of lenvatinib with immunotherapy.

Methods

Search Strategy

We conducted a thorough literature search of studies published between 2014 and 2025, focusing particularly on recent investigations evaluating the efficacy and safety of lenvatinib in treating non-viral aHCC. Additionally, we compared its effectiveness to that of immunotherapy and examined the current challenges faced in the clinical management of HCC. The literature search was conducted through PubMed, MEDLINE (Medical Literature Analysis and Retrieval System Online), Google Scholar, Web of Science and Science Direct using the keywords: “advanced”, “hepatocellular carcinoma”, “non-viral”, “etiology”, “Lenvatinib”, “immunotherapy”, “management”, “challenges”, and “clinical studies” with the filters “humans” and “English language”. Our search strategy and review objectives were well-defined, allowing us to gather information solely from literature that aligned with our aims, as determined by their titles and publication years. Additionally, we performed a manual search of completed and ongoing clinical trials related to lenvatinib in non-viral aHCC registered on www.clinicaltrials.gov. The preliminary search of the electronic database yielded 98 papers. After a comprehensive screening and assessment focused on the study titles, non-viral aHCC cases, comparison of lenvatinib monotherapy with immunotherapy, and main treatment outcomes, including overall survival (OS) and progression-free survival (PFS), 52 papers were selected for inclusion. To enhance transparency, the search strategy, databases, and broad inclusion criteria were outlined. However, in line with the aims and methodological conventions of a narrative review, formal risk-of-bias assessment, reproducibility measures, and quantitative statistical synthesis were not undertaken. Owing to the heterogeneity of the available evidence, clinical outcomes, including survival measures and response rates, were summarized qualitatively based on findings from individual studies.

Study Eligibility Criteria

The review examined articles and clinical studies published as full papers in peer-reviewed journals. The primary emphasis of the investigation was to include studies comparing first-line tyrosine kinase inhibitor (TKI) lenvatinib with immunotherapy in the context of non-viral aHCC, specifically concerning cases associated with MASH/MASLD etiologies. We included systematic reviews, meta-analyses, and clinical studies focusing on the effects of lenvatinib in patients with non-viral aHCC, either as a standalone treatment or in combination with other drugs. Additionally, articles that compared lenvatinib to immunotherapy were included. The titles and abstracts of each article were meticulously reviewed to avoid any duplication. Articles reporting incomplete findings and studies related to HCC with viral etiology and conditions other than HCC were excluded. Clinical studies of any design were included without any restrictions. However, details of the study phase, intervention, and treatment outcomes reported in the published literature were verified by the clinical trial registry IDs on www.clinicaltrials.gov. The search strategy is summarized in Table 1.

**Table 1:** Summary of the search strategy aHCC: advanced hepatocellular carcinoma; ICI: immune checkpoint inhibitor; MEDLINE: Medical Literature Analysis and Retrieval System Online

Items	Specification
Date of search (specified to date, month, and year)	December 29, 2023, to May 28, 2025
Databases and other sources searched	PubMed, MEDLINE, Google Scholar, Web of Science, Science Direct, and ClinicalTrials.gov
Search terms used (including MeSH and free text search terms and filters)	“advanced”, “hepatocellular carcinoma”, “non-viral”, “lenvatinib”, “immunotherapy”, “management”, “challenges”, and “clinical studies”
Timeframe	2014-2025
Inclusion and exclusion criteria (study type, language restrictions, etc.)	Studies published in English from 2014 to 2025. Relevant randomized controlled trials, observational studies, and real-world evidence comparing lenvatinib and ICIs in non-viral aHCC were included.
Selection process (who conducted the selection, whether it was conducted independently, how consensus was obtained, etc.)	Initial screening was performed independently by two reviewers based on title and abstract. Full-text articles were retrieved and assessed for eligibility. Any discrepancies were resolved through discussion and consensus.
Any additional considerations, if applicable	NA

## Review

Molecular pathways involved in the development of non-viral aHCC

**Figure 1:**
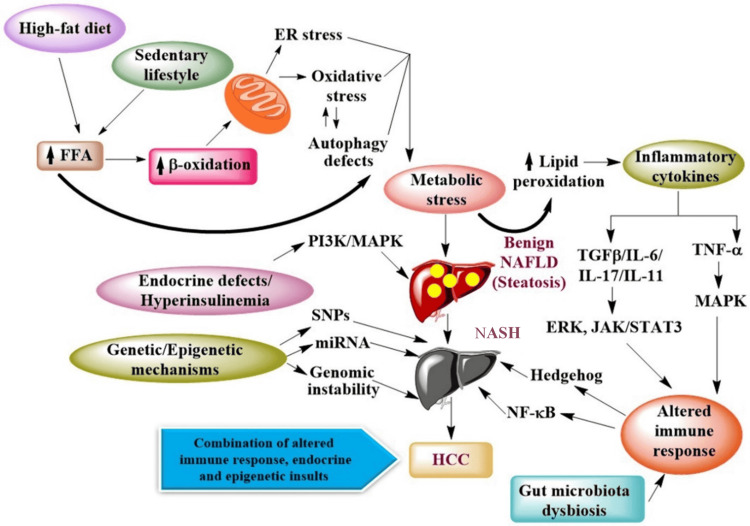
Molecular mechanism involved in the progression of non-viral aHCC aHCC: advanced hepatocellular carcinoma; FFA: free fatty acid; ER: endoplasmic reticulum; MAPK: mitogen-activated protein kinase; PI3K: phosphatidylinositol 3-kinases; NAFLD: non-alcoholic fatty liver disease; TGF-β: transforming growth factor β; IL: interleukin; TNF-α: tumour necrosis factor-alpha; ERK: extracellular receptor kinase; JAK: Janus kinase; STAT: signal transducer and activator of transcription; NASH: non-alcoholic steatohepatitis; NF-κB: nuclear factor kappa-light-chain-enhancer of activated B-cells; SNPs: single nucleotide polymorphisms; miRNA: micro RNA; HCC: hepatocellular carcinoma Image adapted from: Raza et al., 2019 [23]; licensed under CC BY 4.0 Attribution 4.0 International Deed (https://creativecommons.org/licenses/by/4.0/)

The pathophysiology of HCC involves several factors, including genetic or epigenetic modifications, along with complex alterations in the immune signaling pathways, energy metabolism, cellular growth, and proliferation. These alterations in cells eventually lead to the development of HCC, triggered by a series of events, viz., inflammation, hepatocyte injury, and fibrosis (Figure 1) [23,24].

Single-nucleotide polymorphisms (SNPs) are the genetic variations that contribute to MASLD and its subsequent progression to advanced fibrosis [25]. On the other hand, the development of MASH-associated HCC is a consequence of a specific gene, i.e., patatin-like phospholipase domain-containing protein 3 (*PNPLA3*) polymorphism. The risk of developing HCC is three times greater in patients with the *PNPLA3* gene variant [22]. In addition, correlation of high-fat diet, obesity, and diabetes to MASLD/MASH and subsequently to HCC indicates a molecular association between cell cycle regulation and energy metabolism within the hepatocytes, which could be an underlying mechanism responsible for the development of MASH-related HCC. The preclinical investigations reported that oxidative stress, abnormal lipid metabolism, endoplasmic reticulum (ER) stress, and mitochondrial dysfunction might play a role either independently or collectively in the development of MASH-associated HCC [24,26]. The inhibitor of nuclear factor-kB kinase (IKK) dependent nuclear factor-kB (NF-kB) signaling in hepatocytes inhibits the progression of hepatic cancer and promotes the survival of hepatocytes [27]. Subsequent release of inflammatory cytokines through reactive oxygen species (ROS) and lipid peroxidation promotes tumor necrosis factor-alpha (TNF-α)-mediated hepatocellular carcinogenesis via activation of hepatic progenitor cells and interleukin-6 (IL-6) mediated activation of signal transducer and activator of transcription 3 (STAT3) that promotes cell proliferation and inhibits apoptotic pathways, ultimately resulting in the progression of MASH-related HCC [23].

Current challenges in the management of non-viral aHCC

HCC treatment turned out to be a significant challenge in the therapeutic landscape of oncology. The underlying etiologies, either viral or non-viral, play a crucial role in determining the biological characteristics of the tumor, as well as its response to the treatment [16]. The preliminary findings suggest less therapeutic efficacy of immunotherapy in MASLD-HCC patients, which may be attributable to gut dysbiosis, altered immune microenvironment, and MASLD-associated other pathophysiological factors [19]. The IMbrave150 trial outcomes indicate limited efficacy of atezolizumab/bevacizumab combination compared to sorafenib in terms of OS among aHCC patients with non-viral etiologies [28]. Furthermore, subgroup analyses from phase 3 trials (KEYNOTE-240, IMbrave150, and CheckMate-459) suggest that ICIs may be more effective in viral HCC than in non-viral HCC; however, these findings remain exploratory [29]. Another meta-analysis also reported less efficacy of ICIs among patients with non-viral HCC, whereas the therapeutic efficacy of anti-vascular endothelial growth factor (VEGF) drugs or TKIs remains unaffected regardless of underlying etiology [30,31]. Further, a retrospective study reported a lower disease control rate (DCR) among HCC and MASLD-related cirrhosis patients compared to those from the non-MASLD group (64% vs. 89%) [32].

The phase III HIMALAYA trial demonstrated that the STRIDE regimen (single-dose tremelimumab plus durvalumab) significantly improved overall survival compared with sorafenib in unresectable hepatocellular carcinoma, with sustained long-term benefits observed across clinically relevant subgroups, suggesting broadly consistent treatment effects across etiological categories [33]. However, there remains limited prospective and retrospective evidence directly comparing the efficacy of durvalumab plus tremelimumab versus lenvatinib specifically in non-viral aHCC.

Notably, evidence suggests that these variations in the clinical efficacy of ICIs depending on the etiological cause of HCC could be partially attributed to inherent changes in the tumor microenvironment (TME) [34]. Preclinical studies have reported that despite an increase in CD8+ PD-1+ cells in the mice model with MASH-associated HCC, administration of anti-PD1/PD-L1 immunotherapy does not effectively reduce tumor burden, size, or number; instead, it exacerbates incidences of liver fibrosis [29]. Further, to determine whether the preclinical findings can be extrapolated to humans, Agarwal and colleagues identified comparable profiles of CD8+ PD-1+ T cells in the liver samples of patients with MASH/MASLD-associated HCC [35]. Both the preclinical and clinical findings suggest that ICIs do not possess significant success among MASH-HCC patients compared to those with viral HCC. Therefore, it is important to optimize the therapeutic strategies based on the characteristics of HCC patients.

Effectiveness of lenvatinib in patients with non-viral aHCC

Lenvatinib is a potent MKI that specifically targets VEGF receptor (VEGFR), fibroblast growth factor receptors (FGFR), platelet-derived growth factor receptor alpha (PDGFRα), KIT proto-oncogene receptor tyrosine kinase (KIT), and REarranged during Transfection (RET) [36].

**Figure 2:**
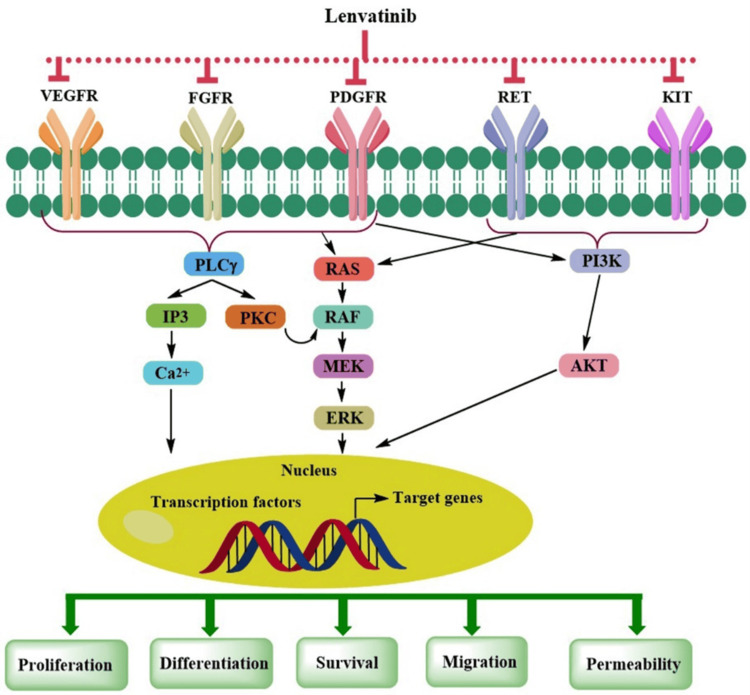
Lenvatinib targeted signaling pathways in aHCC aHCC: advanced hepatocellular carcinoma Image adapted from: Tao et al., 2023 [37]; licensed under CC BY-NC 3.0, Attribution-NonCommercial 3.0 Unported Deed (https://creativecommons.org/licenses/by-nc/3.0/)

Its primary function is to suppress the activation of phospholipase Cγ (PLCγ), Rat Sarcoma/Rapidly Accelerated Fibrosarcoma/ Extracellular signal-Regulated Kinase (RAS/RAF/ERK), and Phosphoinositide 3-Kinase/Protein Kinase B (PKB) PI3K/AKT pathways (Figure 2). Additionally, it suppresses tumor angiogenesis, resulting in nutrient deprivation and hypoxia, thus hindering tumor growth and promoting apoptosis in tumor cells. It also plays a significant role in preventing RET phosphorylation and limiting the activity of proto-oncogene c-KIT, consequently decreasing cell proliferation. Moreover, lenvatinib can influence tumor immune microenvironment (TIME) by reducing the proportion of myeloid-derived suppressor cells (MDSCs) and other negative-regulatory immune cells while increasing the number of activated CD8 T cells [37].

Evidence of Lenvatinib Monotherapy in Non-Viral aHCC

The pivotal REFLECT trial demonstrated the therapeutic efficacy of lenvatinib in advanced HCC, leading to a shift in clinical practice and establishing it as a viable first-line treatment option for aHCC [17]. In a multicenter retrospective analysis, lenvatinib monotherapy showed superior OS in patients with MASLD-associated HCC compared to those with other etiologies (22.2 vs. 15.1 months) [16]. Further, a retrospective study on lenvatinib reported better PFS in the MASLD/MASH-associated HCC group than the viral/alcohol HCC group (9.3 vs. 7.5 months). However, the study reported numerically better but statistically nonsignificant differences in OS between the groups (20.5 vs 16.9 months) [30]. A subgroup analysis of a retrospective observational REFLECT trial demonstrated superior efficacy of lenvatinib than sorafenib in terms of PFS outcomes among patients with alcohol related aHCC. A retrospective study in 67 aHCC patients treated with first-line lenvatinib demonstrated better response rate, OS, and PFS in the MASH/alcohol associated unresectable HCC group than those with HCC with viral etiologies [20]. Studies have demonstrated that lenvatinib was well tolerated, with the most frequently reported adverse events (AEs) including fatigue, diarrhea, foot-skin reactions, and decreased appetite. These AEs were mild and manageable [16,20]. Table 2 depicts multiple clinical studies that highlight the successful use of lenvatinib in the treatment of non-viral aHCC, establishing it as a promising therapeutic option for patients suffering from this condition.

**Table 2:** Clinical evidence of lenvatinib in non-viral aHCC aHCC: advanced hepatocellular carcinoma; HBV: hepatitis B virus; HCV: hepatitis C virus: HR: hazard ratio; MAFLD: metabolic dysfunction-associated fatty liver disease, MASH: metabolic dysfunction-associated steatohepatitis; ORR: objective response rate; OS: overall survival; PFS: progression-free survival

Author, year	Study design	Study population	Intervention (Dose)	Outcomes
Rimini et al., 2021[16]	Multicenter retrospective study	1232 HCC patients (median age 75 years) with HCV (n = 453), HBV (n = 268), MASH (n = 236) and other (n = 275) etiologies	Lenvatinib (8 mg in patients weighing <60 kg or 12 mg in those weighing ≥60 kg once daily)	Lenvatinib-treated patients with MASH-HCC demonstrated longer median OS (22.2 vs. 15.1 months), (HR 0.69; P < 0.0006) and PFS (7.5 vs. 6.5 months), (HR 0.84; P < 0.0436) than patients with other etiologies.
Hiraoka et al., 2021[30]	Retrospective, multicenter study	103 patients (median age 75 years) with MASLD/MASH associated HCC and 427 patients (median age 73 years) with viral/alcohol etiology related HCC	Lenvatinib (8 mg in patients weighing <60 kg or 12 mg in those weighing ≥60 kg once daily)	PFS was significantly better in the MASLD/MASH group than the viral/alcohol group (9.3 vs. 7.5 months), while no significant difference was observed in OS (20.5 vs 16.9 months) between both groups.
Tomonari et al., 2021[20]	Retrospective, observational study	67 patients (median age 71 years) with unresectable aHCC consisting of ^‡^HCV (n = 26), HBV (n = 19), alcohol (n = 11) and MASH (n = 11) cases	Lenvatinib (4 mg/day for patients weighing <40 kg, 8 mg/day for those weighing <60 kg or 12 mg/day for patients weighing ≥ kg)	ORR (59.1 vs. 46.7%) and PFS (13.7 vs. 6.6 months), (HR 0.324; P < 0.01) were significantly higher in non-viral group (MASH/alcohol) than the viral group (HBV/HCV) respectively.
Singal et al., 2023 [38]	Retrospective cohort study	67 patients (median age 62.3 years) with MASH-related HCC	Lenvatinib (12 mg once daily)	PFS rate – 3 months (90%), 6 months (77%) and 12 months (43%); OS rate – 3 months (100%), 6 months (93%) and 12 months (66%)
Shimose et al., 2023[39]	Retrospective study	320 non-viral HCC patients (median age 65 years) with ^||^MAFLD (n = 155) and non-^||^MAFLD (n = 165) cases	Lenvatinib (8 mg in patients weighing <60 kg or 12 mg in those weighing >60 kg once daily)	The OS rate (21.1 vs. 15.1 months) was significantly higher in ^||^MAFLD group than the non-^||^MAFLD group
Casadei-Gardini et al., 2022[40]	Retrospective multicenter study	1325 aHCC patients (median age 72 years) with MASH/MASLD, HBV, HCV and etiologies	Lenvatinib (8 mg in patients weighing <60 kg or 12 mg in those weighing ≥60 kg once daily)	Treated patients showed median OS and PFS of 16.1 and 6.3 months respectively. The MASLD/MASH related etiology was independently associated with good prognosis.
Hatanaka et al., 2021[41]	Multicenter retrospective study	139 aHCC patients (median age 72 years) with viral (n = 84) and non-viral (n = 55) etiologies	Lenvatinib (8 mg in patients weighing <60 kg or 12 mg in those weighing ≥60 kg once daily)	Lenvatinib demonstrated no significant difference in OS and PFS among patients between viral and non-viral HCC
Rimini et al., 2022[42]	Multicenter retrospective study	MASH/MASLD related-aHCC patients treated with lenvatinib (n = 569) and atezolizumab + bevacizumab (n = 190)	-	Lenvatinib treated patients with MASH/MASLD-related aHCC demonstrated significantly better OS (21.2 vs. 12.2 months) and PFS (7.9 vs. 5.1 months) compared to those treated with atezolizumab + bevacizumab
Sacco et al., 2025 [43]	Multicenter cohort study	378 HCC patients Adults with unresectable or advanced hepatocellular carcinoma	-	Treated patients showed median OS and PFS of 21 and 9 months, respectively; outcomes were comparable between MASH/MASLD- and alcohol-related HCC, with prognosis driven by Child–Pugh class and tumor stage rather than etiology.

Safety of Lenvatinib

As observed in the RELEVANT European real-world study, lenvatinib demonstrated a manageable safety profile in patients with MASH-associated HCC, with other toxicity (46%), fatigue (32.5%), and decreased appetite (31.9%) being the most common adverse events [40]. In large Japanese cohorts, including post-marketing surveillance and multi-centre retrospective studies, the most frequently reported AEs were hypothyroidism, hypertension, proteinuria, decreased appetite, fatigue, and diarrhea, with no new safety signals identified in non-viral HCC patients [30,36]. Global real-world data, encompassing cohorts from Europe and Asia, similarly reported no increase in incidence or severity of AEs in MASH-associated HCC, reinforcing the consistency of lenvatinib’s safety profile across populations. AEs remained in line with those reported for viral HCC. These events are typically mild to moderate in severity and can often be managed with dose interruptions, reductions, or supportive care. Serious AEs have occurred but are generally predictable and not unique to lenvatinib, with no new safety signals identified in long-term or large post-marketing studies. 

Various retrospective real-world studies have demonstrated that lenvatinib exhibits a safety profile comparable to other approved first-line systemic therapies for aHCC, with no significant increase in treatment-related AEs relative to ICI-based combinations or sorafenib [31,42,44,45].

Notably, a recent multi-center propensity score-matched analysis of patients treated with lenvatinib demonstrated no significant difference in AE rates between MASH- and alcohol-related HCC cohorts, suggesting that lenvatinib’s safety profile is consistent across these non-viral etiologies. These findings support the absence of distinct etiology-specific toxicity patterns for lenvatinib in non-viral HCC populations [43]. However, future research involving larger, etiology-stratified cohorts is needed to reach definitive conclusions.

Efficacy of Lenvatinib vs. Immunotherapy in Non-Viral aHCC

Patients diagnosed with MASLD-related HCC showed enhanced OS rates following treatment with lenvatinib, whereas the outcomes were less favorable after receiving ICI therapy. However, ICI therapy demonstrated effectiveness for HCC associated with viral infections, while it proved ineffective against non-viral HCC cases [21]. Recently, a retrospective study highlighted the promising outcomes of lenvatinib in the treatment of HCC patients with MASH/MASLD-associated etiology, leading to an improved OS (21.2 vs. 12.2 months) and PFS (7.9 vs. 5.1 months) when compared to atezolizumab plus bevacizumab [42]. Similarly, a retrospective multi-centre study conducted by Casadei-Gardini and Colleagues (2023) reported that patients with MASH/MASLD-associated HCC who received lenvatinib had longer OS compared to those who received atezolizumab and bevacizumab combination therapy [31]. The prognostic and predictive role of neutrophil-to-lymphocyte ratio (NLR) as a potent biomarker has been extensively studied among cancer patients treated with immunotherapy. Notably, a retrospective study reveals that MASH/MASLD patients treated with lenvatinib had a lower NLR in comparison to the patients treated with atezolizumab and bevacizumab (32.5% vs. 41.5%) [42]. A recent retrospective study demonstrated that the objective response rate (ORR) was comparable between patients with MASLD receiving lenvatinib (26.1%) and those treated with atezolizumab and bevacizumab combination (22.7%). Similarly, the DCR was also comparable between lenvatinib (77.2%) and the atezolizumab and bevacizumab combination therapy (70.8%). However, patients treated with lenvatinib experienced a significantly longer median OS of 1364 days compared to 663 days for those receiving atezolizumab and bevacizumab combination [46].

The immunogenicity of viral etiology-associated HCC makes ICIs more potent in their action due to the favorable TME, while, on the other hand, MASH-associated HCC cases represent an accumulation of exhausted CD8+PD1+T cells in the TME [29]. In contrast, lenvatinib monotherapy possesses the ability of immune editing, even in cases when TME has already developed an excluded immune status. Additionally, lenvatinib-mediated potential inhibition of VEGFR and FGFR results in immune modulatory effects within the tumor [47]. These outcomes suggest that targeting both VEGF and fibroblast growth factor (FGF) signaling with lenvatinib could offer more benefits against non-viral aHCC compared to immunotherapy. In recent clinical biomarker studies, partial blockade of myeloid cell inflammation and VEGF-mediated Treg proliferation have been observed in HCC patients undergoing combined immunotherapy [47-49]. The growing evidence suggests that lenvatinib offers significant benefits as a first-line therapy for the effective management of non-viral aHCC, particularly with MASH/MASLD etiologies. However, the main limitation lies in the lack of data from prospective randomized controlled trials (RCTs), as most of the existing studies are retrospective in nature. These studies involve small sample sizes, making them prone to inherent biases, and the population examined across different treatment groups may not be adequately comparable. Therefore, large-scale RCTs are warranted to investigate the efficacy of lenvatinib in non-viral etiologies compared to immunotherapeutic agents. Future research will certainly provide some insights and help in identifying potential management strategies for non-viral aHCC.

Clinical Potential of Lenvatinib in Combination Therapy

**Figure 3:**
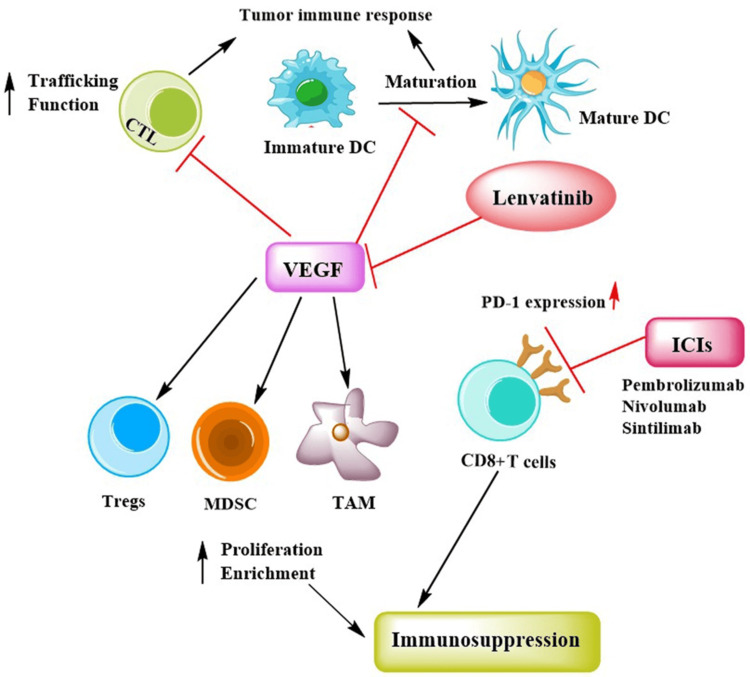
Synergistic effects of lenvatinib and ICIs in restoring VEGF-mediated immunosuppression ICI: immune checkpoint inhibitor; VEGF: vascular endothelial growth factor; CTL: cytotoxic T lymphocyte; DC: dendritic cell; PD-1: programmed cell death protein 1' TAM: tumor-associated macrophage; MDSC: myeloid-derived stem cell; Tregs: regulatory T cells Image adapted from: Zhang et al., 2022 [52]; licensed under CC BY 4.0 Attribution 4.0 International Deed (https://creativecommons.org/licenses/by/4.0/)

Currently, researchers are exploring lenvatinib-based combination therapies to optimize the therapeutic response for the effective management of non-viral aHCC [50,51]. The anti-angiogenic potential of lenvatinib provides complementary benefits when combined with other drugs such as ICIs to effectively eliminate cancer cells. It exerts immunomodulatory effects through promoting maturation of dendritic cells, reversing hypoxia-induced immunosuppressive effect in tissue, and enhancing T cell trafficking and function (Figure 3) [52,53].

In recent studies, lenvatinib in combination with ICIs demonstrated significant therapeutic potential in aHCC [54,55]. Several preclinical studies have reported that the combination of lenvatinib and PD-1 inhibitors results in tumor regression via activation of immune pathways, inhibition of tumor growth factor-β (TGF-β) signaling, decreased infiltration of regulatory T-cells within the tumor, and enhanced interferon-gamma (IFNγ) signaling [56-58]. In a retrospective study, lenvatinib combined with anti-PD1 antibody (nivolumab) exhibited significantly higher OS (22.9 vs. 10.3 months), PFS (7.5 vs. 4.8 months), and ORR (45.0 vs. 23.4%) in patients compared to lenvatinib monotherapy [59]. A retrospective controlled study investigated the therapeutic efficacy of lenvatinib in combination with transarterial chemoembolization (TACE), and their findings revealed that lenvatinib plus TACE exhibited superior efficacy, resulting in higher ORR (64.0 vs. 33.3%) among HCC patients as compared to the TACE plus sorafenib combination [60]. This enhanced efficacy of lenvatinib can be attributed to its stronger affinity for VEGFR than sorafenib [61].

Across three retrospective cohorts encompassing viral, non-viral, MASH-, and MAFLD-related HCC, lenvatinib consistently demonstrated meaningful survival benefit, with comparable OS and PFS across etiologies [41], high early disease control in MASH-HCC [38], and significantly prolonged OS in MAFLD-associated HCC versus non-MAFLD disease [39], collectively supporting its efficacy irrespective of underlying liver disease.

Limitations

The included clinical trials were neither specifically designed nor statistically powered to evaluate etiology-specific differences in treatment response. Consequently, subgroup analyses stratified by underlying etiology must be interpreted with caution, as they are exploratory in nature and hypothesis-generating rather than definitive.

## Conclusions

Lenvatinib has emerged as an important therapeutic option in the management of non-viral aHCC. The dual antiangiogenic and immunomodulatory mechanisms of lenvatinib provide a strong biological rationale for its enhanced activity in non-viral HCC, including MASH/MASLD-associated disease, where real-world evidence suggests favorable clinical outcomes, such as prolonged progression-free survival and higher objective response rates.

Based on retrospective and exploratory subgroup analyses, lenvatinib remains an important therapeutic option in the management of non-viral aHCC. Importantly, emerging data indicate that lenvatinib-based combinations with immunotherapy may confer survival benefits across etiological subgroups, highlighting its potential role within combination treatment strategies. However, well-designed, large-scale, prospective randomized controlled trials are needed to validate these findings and optimize sequencing, combination, and personalized treatment strategies in aHCC.
